# Highly Fluorinated Barium Titanate Nanoparticle Dispersion for Fabrication of Lithographically Patterned Thin Films

**DOI:** 10.3390/ma12244045

**Published:** 2019-12-05

**Authors:** Youngtae Kim, Heejin Kim, Hyun-Taek Oh, Sangwon Kim, Jin-Kyun Lee

**Affiliations:** Department of Polymer Science and Engineering, Inha University, Incheon 22212, Korea; hellpo@naver.com (Y.K.); bsfam1234@gmail.com (H.K.); oht3431@gmail.com (H.-T.O.)

**Keywords:** high dielectric constant, barium titanate, fluorinated materials, lithographic patterning

## Abstract

We report the synthesis, characterization, and photopatterning of high-*k* inorganic nanoparticles that are covered with highly fluorinated carboxylic acid and, as a result, are solution-processable in fluorous liquids. Barium titanate (BTO) nanoparticles, 7–8 nm in diameter, were prepared under solvothermal conditions and were surface-modified with perfluoroalkyl ether-type carboxylic acid molecules via ligand-exchange reactions. Thin films with a high dielectric constant (9.27 at 1 kHz) were achieved by spin-coating homogeneous solutions of BTO nanoparticles in a fluorous solvent (HFE-7500). Additionally, electron-beam lithography and photolithography were applied to the thin films of BTO nanoparticles, yielding BTO patterns with scales of 300 nm and 5 μm, respectively. Thus, an approach for a chemically non-damaging solution process of inorganic materials for device implementation was successfully demonstrated.

## 1. Introduction

Designing suitable dielectric materials is deemed essential for various applications, including transistors, passive capacitors, power-storage devices, and actuators. In addition to the dielectric constant, various aspects of gate dielectrics that bridge the active semiconductor layer and gate electrodes in organic field-effect transistors (OFETs) play a crucial role in determining the overall operation and performance of OFETs [1,2]. Notably, the limited voltage range of the power supply available in typical OFET applications (e.g., wearable sensors) necessitates new gate dielectrics with enhanced dielectric constants (*k*), which would allow OFETs to operate at low voltages.

Metal oxides and silicon nitrides, fabricated using plasma-enhanced chemical vapor deposition [3] and sputtering [4], have been studied as gate dielectrics in OFETs. While the common tools for oxide fabrication often require an expensive, complex vacuum process and/or elevated temperatures, methods for fabricating high-*k* dielectrics amenable to the solution-processing protocol are highly desirable for OFET gate dielectrics considering inexpensive, low-end applications of OFETs. Solution-based processes, such as spin-coating, spraying, and printing, are employed to deposit self-assembled monolayers [5], inorganic oxides [6], and polymer thin films [7,8,9]. However, polymers typically have dielectric constants far lower than those of inorganic materials [10]. Additionally, the gate leakage problem and pinhole formation usually set the lower limit for the film thickness of polymer gate dielectrics, making it difficult to realize the large gate capacitance required for low-voltage OFET operation [9,11].

Nanocomposites that consist of inorganic nanoparticles and organic hosts have been studied for the purpose of achieving a high gate dielectric constant and processability [12]. Often, the surface modification of inorganic particles that involves ligand or polymer attachment at the particle surface is implemented to endow them with a high affinity to a matrix [13,14], and the resulting good dispersion makes it feasible to fabricate dielectric thin films using various solution-processing methods. Thin-film composites that consist of high-*k* ceramic nanoparticles, such as BaTiO_3_ (BTO) and TiO_2_, have been characterized and implemented as gate dielectric materials in OFETs [6,15,16]. The dielectric constants of composites exhibit significant dependence on the dielectric constants and loading fractions of the ceramic constituents in the composites. However, various studies have revealed that the effective dielectric constants of nanocomposites are influenced by the homogeneity of the dispersion in the composites, which depends on factors such as filler size/shape and ligand chemistry [17,18].

**Scheme 1:**
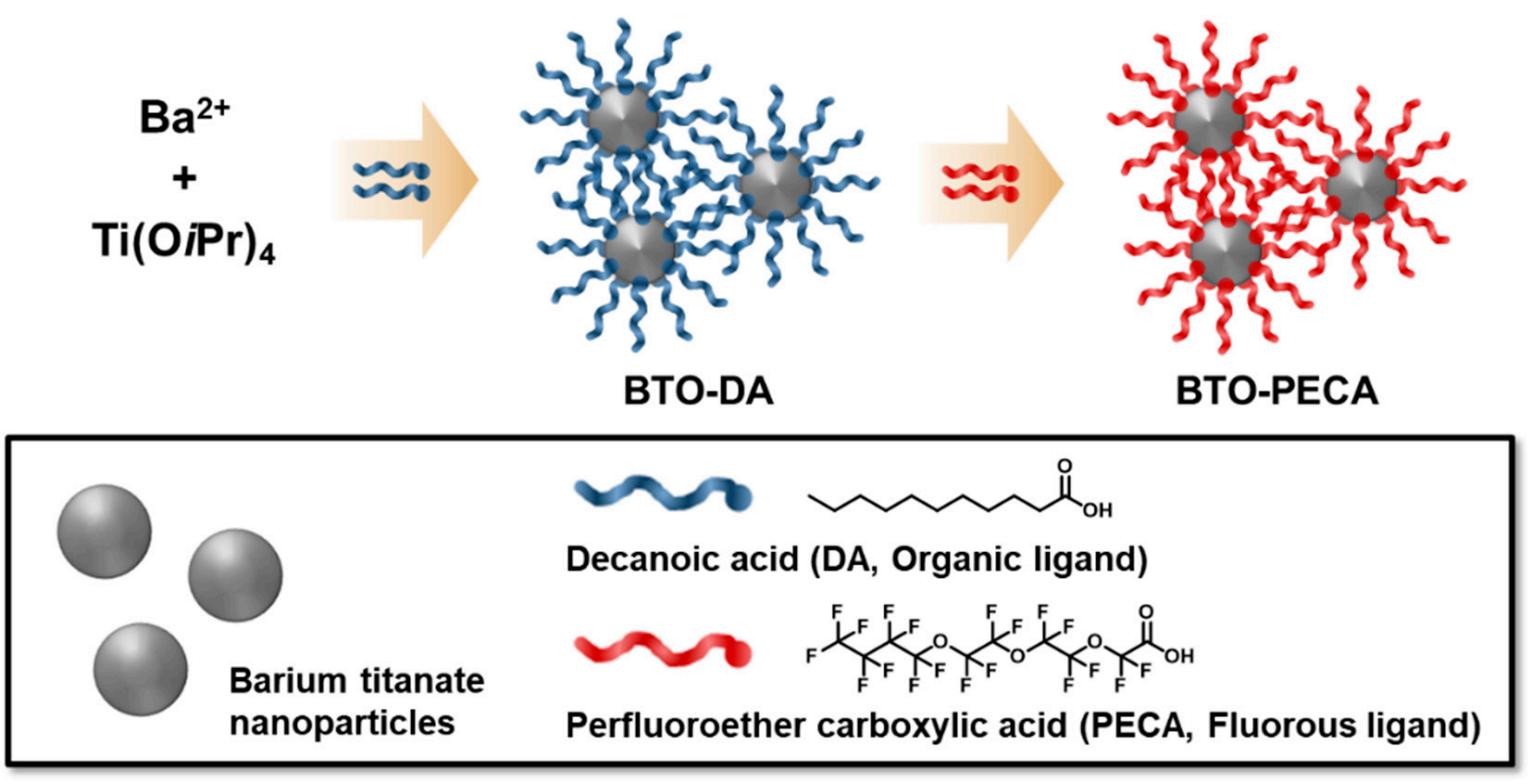
Synthesis of decanoic acid-capped barium titanate (BTO-DA) and perfluoro-3,6,9-trioxatridecanoic acid-capped barium titanate (BTO-PECA) via the solvothermal method and the ligand-exchange reaction.

Electronic devices generally consist of stacked layers with distinct functionalities fabricated via multiple processing steps, and the integrity of one layer may be compromised depending on its compatibility with the rest of the processing steps. For example, in many cases, a layer of organic semiconductor material tends to be damaged upon subsequent solution processing steps. Chemical orthogonality afforded by fluorous material chemistry allows the fabrication of multiply stacked layer structures without undermining the intended functionality of the established organic materials [19]. High-*k* nanocomposites with such chemical orthogonality can be realized by encapsulating nanoparticles with fluorous organic shells [20]. In 2017, we modified the surface of BTO nanoparticles (diameter of approximately 80–100 nm) with fluorine-containing moieties, achieved good dispersion in fluorous solvents, and fabricated dielectric layers of *k* = 21.5 (at 1 kHz) [21]. While this value is higher than what is achievable with general polymer dielectrics, one method to further reinforce the dielectric properties of composite layers involves the bimodal mixing of particles of disparate sizes, which allows an even higher filler packing density [22]. Considering the complex size dependence of the BTO particle structure and the dielectric constant [22,23,24], in this study, we investigated small BTO particles prior to mixing with larger BTO particles (80–100 nm). BTO nanoparticles (7–8 nm) were synthesized and surface-modified with fluorous ligands, as shown in Scheme 1. Their dispersions in fluorous solvents were prepared successfully, characterized, and used to fabricate thin-film dielectric layers via simple spin-coating. The direct photopatterning of BTO thin films was achieved via electron-beam (e-beam) lithography and photolithography.

## 2. Materials and Methods

### 2.1. Synthesis of Decanoic Acid-Capped BTO Nanoparticles (**BTO-DA**)

Among the various chemical methods for synthesizing nanoparticles (e.g., sol-gel [25], hydrolysis of metallic precursors [26]), the nonhydrolytic synthesis of BTO nanoparticles with surface capping ligands was employed in this study [27]. BTO nanoparticles were prepared as described by Chen et al. [27]. Benzyl alcohol (Sigma–Aldrich, St. Louis, MO, USA) (20 mL) was added to 0.74 g of metallic Ba (Strem, Newburyport, MA, USA) in a round-bottom flask. The mixture was stirred at 80 °C until the metallic Ba was clearly dissolved and the solution became transparent, with a pale yellow color. Then, titanium (IV) isopropoxide (1.53 g, Sigma–Aldrich, St. Louis, MO, USA) was syringe-injected into the solution at room temperature, followed by stirring at 30–50 °C until the formation of white precipitates. The precipitates were added to ligand solutions that consisted of oleylamine and a 2.5-fold excess of decanoic acid (TCI, Tokyo, Japan). The reaction proceeded at 320 °C and was terminated after 24 h [28]. The resulting **BTO-DA** particles were recovered via centrifugation (4000 rpm, 5 min) after adding polar solvents (e.g., ethanol, acetone). The particles were washed for several cycles via re-dispersion in toluene followed by precipitation to remove any organic residue. Approximately 0.31 g of **BTO-DA** was obtained as a light brown powder. The crystal structure of the nanoparticles was examined using X-ray diffraction (XRD) analysis (XPert-PRO MRD, Philips, Amsterdam, The Netherlands). The size of the nanoparticles in the solution was estimated using dynamic light scattering (DLS) (ELS-Z, Otsuka, Hirakata, Japan), and real-space images of the nanoparticles were obtained using a transmission electron microscope (TEM) (CM200, Philips, Amsterdam, The Netherlands).

### 2.2. Ligand-Exchange Reaction of BTO-DA to BTO-PECA (Perfluoro-3,6,9-trioxatridecanoic Acid-Capped BTO Nanoparticles)

While the preparation of nanoparticles capped with fluorous moieties has been achieved through various methods [29,30,31], in this study, BTO nanoparticles with highly fluorinated ligands were obtained using ligand-exchange reactions [32,33]. To impart solubility to the synthesized BTO nanoparticles (**BTO-DA**) in a fluorous solvent, perfluoro-3,6,9-trioxatridecanoic acid (**PECA**) (Fluorochem, Hadfield, UK)—a perfluoroalkyl ether-type ligand functionalized with a carboxylic acid group at the end of the chain—was used to substitute the organic capping ligand surrounding the inorganic nanoparticles. Highly concentrated dispersions of nanoparticles are often recommended for colloidal processing, and one of the methods for attaining a high concentration involves minimizing the thickness of the organic layers surrounding the ceramic nanoparticles [34]. Accordingly, we used **PECA**, which has a relatively low molecular weight, as a colloid stabilizer. **BTO-DA** (0.31 g) and **PECA** (0.93 g) were added to 3-ethoxy-1,1,1,2,3,4,4,5,5,6,6,6-dodecafluoro-2-trifluoromethyl hexane (HFE-7500, 3M, St. Paul, MN, USA) (5 mL), which is a fluorous solvent, followed by stirring at 130 °C for 24 h. The reaction mixture gradually became transparent as the highly fluorinated ligands were bound to the particle surface. After the reaction was complete, the temperature was lowered to room temperature, and the solution was filtered using a syringe filter (Nylon, 0.45 μm). An excess amount of acetone was then poured into the filtered solution to precipitate the BTO particles surrounded by the fluorous ligand molecules (**BTO-PECA**), and the final samples were collected via centrifugation. The washing process was repeated several times using acetone, and 0.28 g of **BTO-PECA** was obtained after vacuum drying. Fourier-transform infrared (FT-IR) spectroscopy (VERTEX 80 V, Bruker, MA, USA) was performed to probe the binding of the ligands to the nanoparticle surface. Thermogravimetric analysis (TGA, STA409PC, Netzsch, Selb, Germany) was performed to monitor the change in the weight of the nanoparticles with temperature increments (10 °C/min).

### 2.3. Layer Preparation for Dielectric-Constant Measurement

Cr-coated glass substrates were cleaned with acetone, methanol, and deionized water for 5 min each using ultrasonication. Then, the substrates were cleaned via ultraviolet (UV)–ozone treatment. The BTO insulator layer on the Cr layer of the substrates was fabricated by spin-coating BTO dispersions (5 wt./vol.%; **BTO-DA** in toluene, **BTO-PECA** in HFE-7500) at 1500 rpm for 1 min. The coated samples were baked and annealed sequentially at 70 and 100 °C for 30 min each. The thickness of the films was measured using a KLA-Tencor surface profiler. Al electrodes (thickness 100 nm) were then thermally evaporated onto the insulator layer through a shadow mask, resulting in the formation of metal–insulator–metal (MIM) structures. The capacitance of the MIM device was measured using an LCR meter (ZM2353, NF Corp., Yokohama, Japan), and the relationship C_i_ = ε_0_*k*/d (ε_0_ represents the permittivity of vacuum, *k* is the dielectric constant, and d represents the film thickness) was used to determine the dielectric constant.

### 2.4. Pattern Fabrication Using Electron-Beam (E-Beam) Lithography and Photolithography

The thin films made of **BTO-PECA** nanoparticles were patterned via e-beam lithography. A **BTO-PECA** dispersion in HFE-7500 (0.05 g/mL) was spin-coated on a Si wafer at 500 rpm, and the substrate was baked at 95 °C for 2 min. The resulting 109 nm thick, solvent-free thin film was subjected to e-beam lithography at an acceleration voltage of 80 keV and a beam current of 0.5 nA using an e-beam exposure tool (NanoBeam NB3, Nanobeam Ltd., Cambridge, UK). E-beams were irradiated to produce 300 nm wide line arrays with controlled periods (600, 800, and 1000 nm). The beam dose was varied systematically from 500 to 3800 μC/cm^2^. The exposed substrate was developed in methoxy-nonafluorobutane (HFE-7100, 3M, St. Paul, MN, USA) for 1 min.

Photolithographic patterning of the **BTO-PECA** nanoparticles was performed by embedding them in an acid-crosslinkable, highly fluorinated polymer binder and a photoacid generator (PAG). The binder polymer was poly(3,3,4,4,5,5,6,6,7,7,8,8,9,9,10,10-heptadecafluorodecyl methacrylate-*r*-glycidyl methacrylate) [**P(FDMA-*r*-GMA)**], which was synthesized via the free-radical polymerization method [35]. First, radical inhibitors added to **FDMA** (Fuxin Hengtong, Fuxin, China) and **GMA** (Sigma–Aldrich, St. Louis, MO, USA) were removed by passing through a short plug of Al_2_O_3_ powder (Sigma–Aldrich, St. Louis, MO, USA). The **FDMA** (4.00 g) and **GMA** (0.75 g) were dissolved in benzotrifluoride (6 mL, Sigma–Aldrich, St. Louis, MO, USA), to which 2,2’-azobisisobutyronitrile (0.048 g, AIBN, Junsei Chemical, Tokyo, Japan) was added. The polymerization mixture in a Schlenk tube was subjected to three freeze–pump–thaw cycles in a N_2_ atmosphere. The sealed tube was heated to 72 °C, with stirring for 8 h. The reaction mixture was cooled to room temperature and poured into n-hexane (Daejung, Siheung-si, South Korea). The resulting polymer powder was recovered via filtration, washed with a copious amount of n-hexane, and finally dried under a reduced pressure to obtain 3.83 g of **P(FDMA-*r*-GMA)** as a white solid.

A dispersion of **BTO-PECA** nanoparticles (0.10 g) was prepared by adding them to a solution of **P(FDMA-*r*-GMA)** (0.30 g) and a PAG (0.015 g, Irgacure^®^ CGI-1907, BASF, Ludwigshafen, Germany) in HFE-7500 (2 mL). It was then spin-coated onto a pre-washed Si wafer at 1500 rpm. The coated substrate was baked at 80 °C for 5 min and exposed to 4.0 J/cm^2^ of 365 nm UV light through a photomask. The exposed wafer was baked again at 90 °C for 5 min and developed in HFE-7500 for 15 s, sequentially. The UV irradiation was performed using a spot exposure-type UV light-emitting diode curing system that emitted 365 nm single-wavelength light (UV LED, SMT UV Technology, Bucheon-si, South Korea). The thicknesses of the fabricated films and patterns after development were measured using a stylus-type thickness profiler (Alpha-Step D-300, KLA-Tencor, Milpitas, CA, USA). The thin films and micropatterns were examined using scanning electron microscopy (SEM) (S4300SE, Hitachi, Tokyo, Japan) after Pt deposition on top of the samples.

## 3. Results and Discussion

### 3.1. Structural and Morphological Properties of BTO Nanoparticles

**Figure 1:**
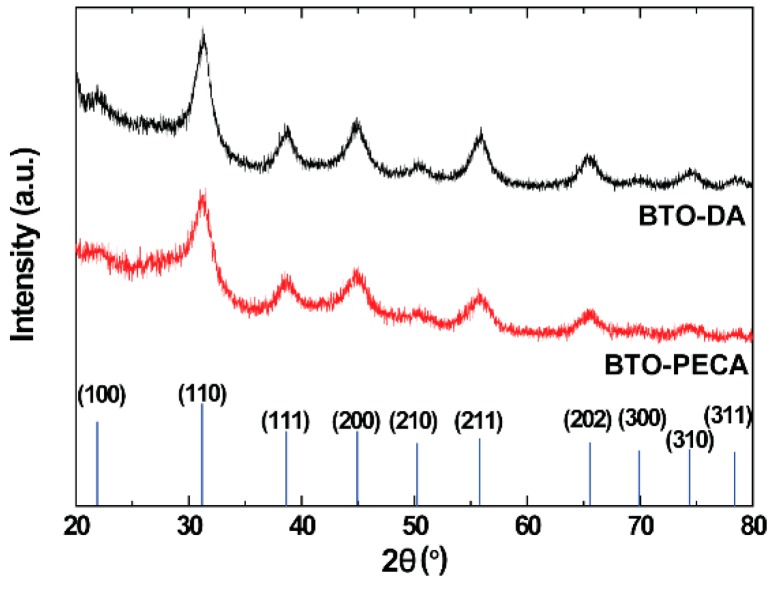
Powder XRD patterns of BTO nanoparticles (BTO-DA and BTO-PECA) compared to the pattern of cubic BTO (JCPDS NO. 31-174).

The XRD patterns of the BTO nanoparticles (**BTO-DA**, **BTO-PECA**) (Figure 1) are consistent with crystalline BTO without the formation of major byproducts such as BaCO_3_ and TiO_2_ that are commonly used as precursors in solid-state reactions to synthesize BTO. The representative peak splitting at 2θ = 45° [i.e., (002), (200)] indicates a tetragonal polymorph [36], but the corresponding peak shown in Figure 1 is apparently too diffuse to make a distinction, presumably owing to the small particle size [37]. On some occasions, broad XRD profiles have been attributed to the coexistence of cubic and tetragonal phases [38]. The average particle sizes of **BTO-DA** and **BTO-PECA** estimated using the Scherrer equation were 3.7 and 5.2 nm, respectively [39]. The similar profiles between **BTO-DA** and **BTO-PECA** suggest that the particles retained their crystal structures through the ligand-exchange reaction. In previous studies, the tetragonal phase of bulk BTO perovskite structures was formed at room temperature, and the cubic phase was stable above the Curie temperature of 130 °C. Notably, Uchino et al. [23] studied the dependence of the crystal structure of BTO on the particle size and observed the transition from tetragonal symmetry to cubic symmetry as the particle size decreased below the critical value of 120 nm. While a broad range of the critical particle size—below which the cubic phase is stable—has been reported, it is common to observe cubic symmetry in BTO nanopowders [36,39].

**Figure 2:**
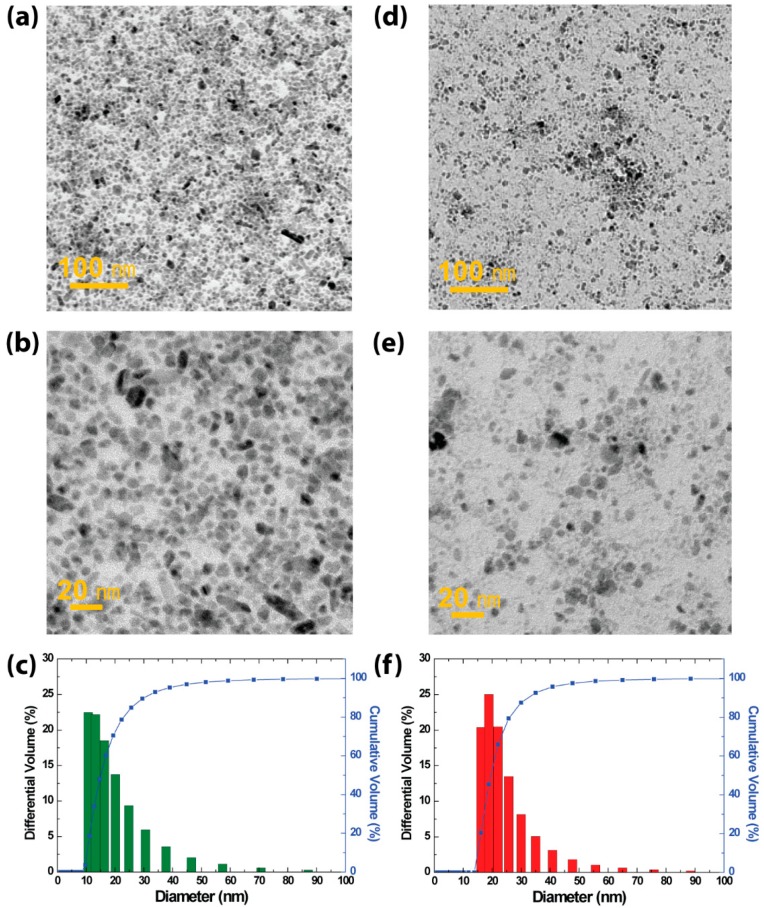
TEM images and particle-size distribution histograms of (**a**–**c**) BTO-DA and (**d**–**f**) BTO-PECA.

Transmission electron microscopy (TEM) was implemented to obtain real-space images of the BTO nanoparticles. While numerous attempts to obtain nonisotropic (elliptical, triangular, rod) nanoparticles with controlled dimensions have been made [40], the TEM micrographs of **BTO-DA** and **BTO-PECA** in Figure 2 show that the BTO nanoparticles were well-dispersed and characterized by mostly spherical morphologies with average particle diameters of 7.8 nm [27]. The aspect ratios of **BTO-DA** and **BTO-PECA** in the TEM images were 1.05 (±0.11) and 1.04 (±0.04), respectively. According to the micrographs, the particle sizes hardly changed after the ligand-exchange reaction. A particle-size analysis revealed that the particle sizes for **BTO-DA** in n-hexane and **BTO-PECA** in HFE-7500 were monomodal, and cumulative histograms indicated that the particle-size range of 0–30 nm accounted for >90% of the nanoparticles (Figure 2c,f). The small shift in the particle size distributions of **BTO-DA** and **BTO-PECA**, observed in Figure 2, may be caused by a small degree of agglomeration, but the **BTO-PECA** solution maintained this homogeneous dispersed state and was solution-processable for thin-film fabrication, as shown later in the paper.

### 3.2. Characterization of BTO Nanoparticle Ligands

**Figure 3:**
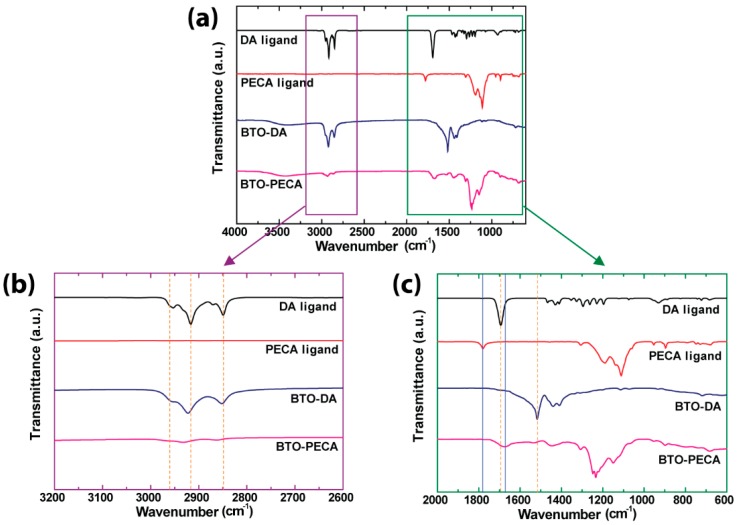
FT-IR spectra of two ligands (DA and PECA) and two surface-modified BTO particles (BTO-DA and BTO-PECA). The wavenumber ranges were (**a**) 4000–600, (**b**) 3200–2600, and (**c**) 2000–600 cm^−1^. The orange dashes and blue lines are for visual guidance.

The surface anchoring of the ligand molecules onto the BTO nanoparticles was examined using FT-IR spectroscopy. Figure 3 shows the spectra of the pristine ligands (**DA**, **PECA**) and surface-modified nanoparticles (**BTO-DA** and **BTO-PECA**). Absorption peaks at 2954, 2923, and 2852 cm^−1^, which are characteristic of alkyl chain stretching of the DA ligand, were clearly observed for **BTO-DA** [41]. For **BTO-PECA**, these peaks were suppressed (but not completely), and absorption peaks of perfluoroalkyl chain stretching at 1307, 1188, and 1110 cm^−1^ emerged. These changes indicate that an effective ligand-exchange reaction, which introduced **PECA** onto **BTO-DA**, was accomplished [20]. The chemical binding of carboxylic acid-functionalized ligands onto metal-oxide surfaces accompanies the conversion of the acid group to the carboxylate form. Therefore, the shift in the characteristic FT-IR absorption peaks suggests the ligand binding on the nanoparticle surfaces [30]. As shown in Figure 3c, the stretching peak at 1695 cm^−1^ corresponding to the carboxylic acid group of the DA ligand was not visible for **BTO-DA**. However, **BTO-DA** exhibited an absorption peak at 1542 cm^−1^, attributable to carboxylates [42]. Similar displacement of an absorption peak was observed when a comparison was made between the **PECA** ligand and **BTO-PECA** (Figure 3c); the **PECA** ligand exhibited a stretching peak at 1780 cm^−1^, whereas the **BTO-PECA** exhibited a carboxylate peak at 1676 cm^−1^. These unique shifts suggest that the carboxylic acid-functionalized ligands were chemically attached to the surface of the BTO nanoparticles via thermal ligand-exchange reactions.

**Figure 4:**
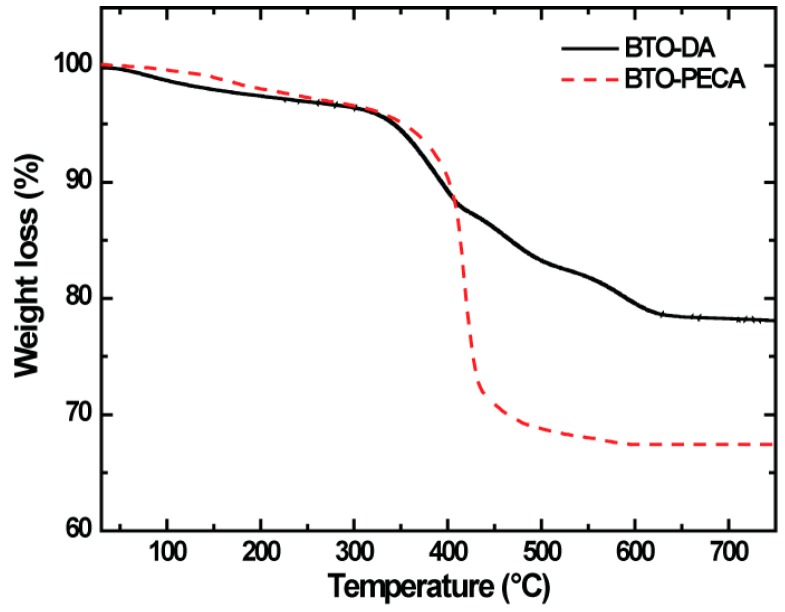
TGA of BTO-DA (solid line) and BTO-PECA (dashed line) in the range from 30 to 750 °C.

The degree of incorporation of the **PECA** ligands onto **BTO-PECA** was investigated using TGA. It is well known that the extent of ligand attachment significantly affects the dispersion characteristics of nanoparticles in a solvent [29,43]. A homogenous dispersion becomes unstable when the coverage of the ligand is insufficient. Therefore, one of the issues with surface modification via ligand association is the reverse, thermally activated ligand dissociation. This reaction leads to the aggregation of the nanoparticles, causing the specimen to lose its distinctive, beneficial characteristics originating from the homogeneous dispersion [44]. According to the TGA results (Figure 4), the onset of significant weight loss occurred above 250 °C, indicative of the robust surface coverage of the nanoparticles by DA and **PECA** ligands. The weight fraction of the attached ligands was quantified based on the TGA weight loss of **BTO-DA** and **BTO-PECA** at high temperatures, presumably pertaining to random decompositions of the organic ligands [29]. The weight loss of 19 wt.% observed for **BTO-DA** translates to the binding of approximately 6 DA ligands per unit area on the nanoparticle surface, assuming unimodal spherical nanoparticles with an average diameter of 7.8 nm (Figure 2). The weight loss (29 wt.%, Figure 4) for **BTO-PECA** originated from the decomposition of **PECA** and unexchanged DA. Assuming a one-to-one substitution of **PECA** ligands for DA through the ligand-exchange reaction, the calculation based on the weight loss suggests that as much as 65% of the DA was replaced with **PECA**. This is consistent with the remnant DA peaks observed in the FT-IR results for **BTO-PECA** (Figure 3). Although the prolonged reaction time enhances the efficiency of the ligand-exchange reactions, it is anticipated that the steric interactions caused by the bulky **PECA** compared to DA would delimit the highest attainable efficiency.

**Figure 5:**
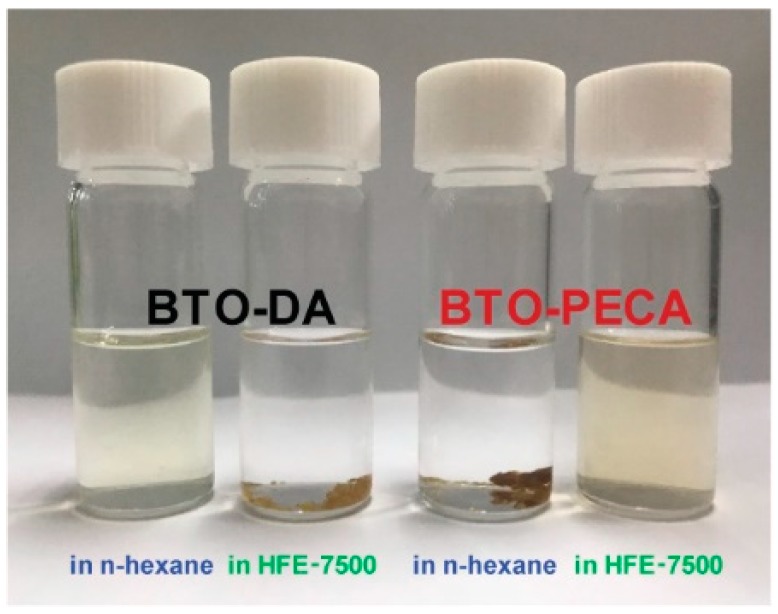
Photographs of vials containing BTO-DA and BTO-PECA in organic and fluorous solvents (hexane, HFE-7500).

The surface-modified **BTO-PECA** exhibited good solubility in the fluorous solvent, which is required for thin-film fabrication using fluorous solution processing. Figure 5 shows vials of **BTO-DA** and **BTO-PECA** in n-hexane and HFE-7500, respectively. The chemistry and miscibility of ligands on nanoparticle surfaces control the preferential localization of particles within the domains of the matrix [45]. While the **BTO-DA** was well-dispersed in n-hexane—an organic solvent compatible with decanoic acid—the macrophase separation observed for **BTO-DA** in HFE-7500 signals unfavorable interaction between the nanoparticles and the fluorous solvent. By contrast, the homogeneous state of the **BTO-PECA** in HFE-7500 indicated dispersion of the nanoparticles in the fluorous solvent, suggestive of effective surface mediation attained via ligand exchange [20]. The homogeneity/heterogeneity of these samples did not change over several months of observation.

### 3.3. Dielectric Constant of BTO-PECA Films

**Figure 6:**
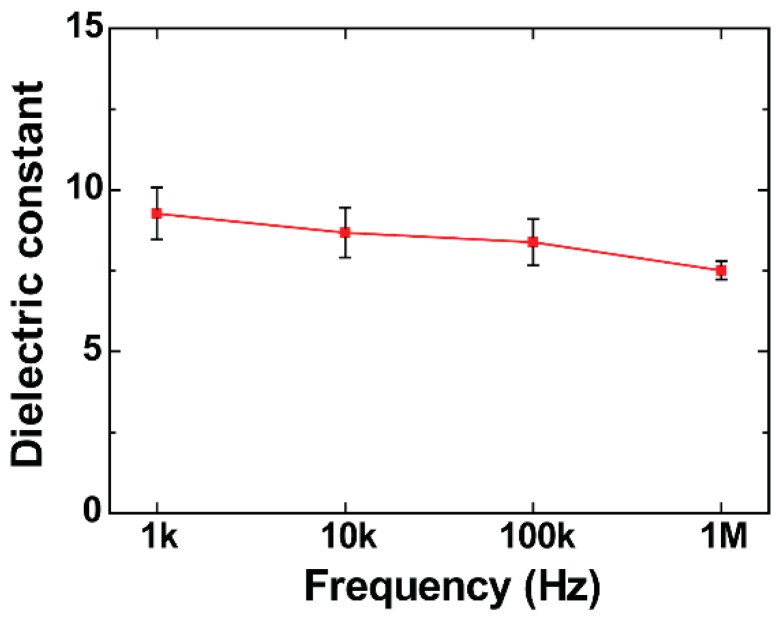
Frequency dependence of dielectric constants of BTO-PECA films obtained via spin-coating.

An MIM structure was fabricated to investigate the dielectric properties of the highly fluorinated inorganic dielectric materials. The solution of **BTO-PECA** in HFE-7500 was spin-coated onto Cr glass to form a 250 nm thick film, which exhibited a dielectric constant of *k* = 9.27 at 1 kHz (Figure 6). This value exceeds those of the fluorinated polymers generally used for capacitors (*k* = 1.9–2.8) [10]. Barium strontium titanate nanoparticles that have a comparable size to **BTO-PECA** (5–8 nm) and are covered by oleic acid molecules have a similar dielectric constant (*k* = 5–6 at 100 Hz) [15]. Notably, these dielectric-constant values for BTO particles in the nanometer regime (<10 nm) are significantly lower than those of BTO composites prepared with larger BTO particles [20,21,22,30,46]. This trend agrees with previous reports on the grain-size dependence of BTO dielectric constants [24,47]. The thickness dependence of the dielectric constant of BTO films has been investigated in the past, and the smaller dielectric constants for the thinner films were attributed mainly to the grain size effect [47,48]. The grain size of the **BTO-PECA** thin films was determined by the nanoparticle size and was expected to be independent of the film thickness.

The Jayasundere–Smith model, one of analytical theories for the 0–3 composite system, accounts for the particle–particle dipolar interaction and is anticipated to be more accurate even at higher filler loading compared to the Kerner model [49]. This model was employed to estimate the dielectric constant of BTO nanoparticles without ligands (*k* = 32.7). This value is significantly higher than that of the ligand-substituted counterparts and comparable to the values attained for BTO particles of similar sizes [50]. Here, the volume fractions of inorganic BTO and the substituting ligands were estimated based on the TGA results. Densities of 1.8 and 6.02 g/cm^3^ were used for the ligands and BTO, respectively. The representative dielectric constant for fluorinated polymers (*k* = 2) was assumed for the ligands. Ligands immobilized onto a rigid filler surface behave differently from those in unbound states and constitute an “interphase region” with unique dielectric characteristics [51]. Considering the nanoscale sizes of the BTO particles studied here, the effect of this interphase region was anticipated to be significant, in agreement with the prediction by Li and coworkers [52]. Therefore, elucidation of dielectric-constant contributions from each phase of the composites would require a detailed analysis accounting for the “interphase” effects, as well as other attributes of the fillers (e.g., spatial distribution and orientation), which is beyond the scope of this paper.

### 3.4. Lithographic Patterning of BTO-PECA

**Figure 7:**
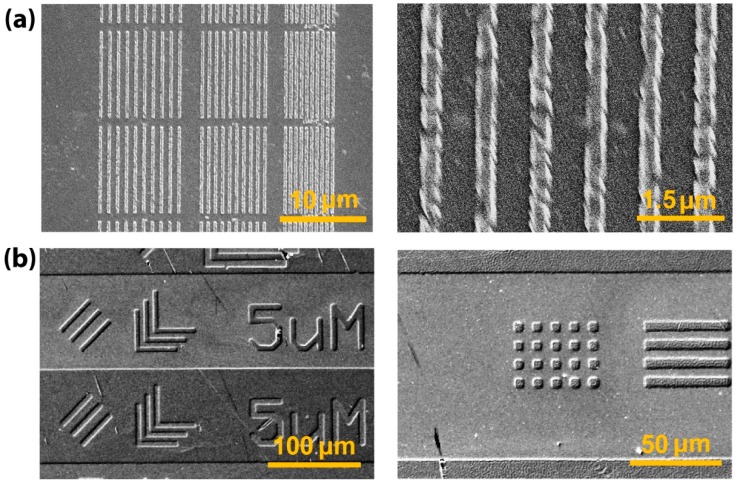
(**a**) SEM images of 300 nm wide BTO-PECA line patterns with periods of 1000, 800, and 600 nm attained using electron-beam (e-beam) lithography. The image on the right is a high-magnification image of the patterns with a 1000 nm period; (**b**) SEM images of nanocomposite films photopatterned with UV exposure.

Two lithographic techniques were implemented independently to generate thin-film patterns of **BTO-PECA**: E-beam lithography and photopatterning with UV exposure. Figure 7a shows SEM images of negative-tone line patterns obtained via e-beam writing. The developed image consists of alternating regions of 300 nm wide lines and spaces (space widths: 700, 500, and 300 nm). The reduced solubility of **BTO-PECA** upon e-beam irradiation is mainly attributed to two competitive mechanisms [53]. C–F bond cleavage on **PECA** induced by high-energy irradiation generated free-radical species, which coupled with each other, forming intermolecular crosslinks. It is also plausible that the **PECA** ligands dissociated from BTO nanoparticles as a result of electronic excitation of the carboxylate group of the PECA ligands, reducing the compatibility of the nanoparticles with the fluorous developing solvent. In this paper, we only present the results obtained at an e-beam exposure dose of 3000 μC/cm^2^ (Figure 7a). The magnified image on the right side of Figure 7a reveals the rough edges of the line patterns, which we believe can be addressed in the future by optimizing the processing conditions.

To fabricate micropatterned structures of high-*k* **BTO-PECA** via UV irradiation, the inorganic nanoparticles were embedded into thin films composed of a crosslinkable binder and a PAG. A random copolymer prepared with **FDMA** and **GMA**, i.e., **P(FDMA-*r*-GMA)**, functioned as a binder as it can form an insoluble polymer network efficiently via acid-catalyzed ring-opening reactions. As a PAG, CGI-1907 was employed, because it could be dissolved in HFE-7500 and liberates strong nonafluorobutane sulfonic acid, whereby the ring-opening reactions are triggered. A homogeneous mixture of **BTO-PECA**, **P(FDMA-*r*-GMA)**, and CGI-1907 in HFE-7500 was spin-coated on a Si wafer to form a 700 nm thick film, and the coated substrate was exposed to 365 nm UV light through a photomask. After a baking step at 90 °C, the substrate was successfully developed in HFE-7500 to produce negative-tone, 5 μm-scale features, as shown in Figure 7b. Notably, the thermal treatments with lower temperatures tended to improve the quality of the resulting patterns. This dependence on the thermal treatment conditions was speculated to be caused by the presence of epoxy-containing glycidyl methacrylate (GMA) components in the polymer binders. GMAs were prone to ring-open and go through a series of reactions at elevated temperatures, potentially causing even the unexposed regions to lose the solubility during the forthcoming development process.

## 4. Conclusions

The solution processability of 7–8 nm barium titanate (BTO) nanoparticles in fluorous solvents was achieved via the modification of the particle surface with the highly fluorinated **PECA** ligand. Thin films were fabricated by spin-coating the **BTO-PECA** dispersion in HFE-7500 and were characterized by a dielectric constant as high as 10.0 at 1 kHz and photopatterning feasibility. Under e-beam lithographic conditions, the **BTO-PECA** films were tailored to produce features with a size of 300 nm without any binder material. Under photopatterning with UV exposure, the nanoparticles embedded into crosslinkable binder films formed 5 μm-scale features via UV-triggered, acid-catalyzed ring-opening reactions. The pattern quality including resolution and line edge roughness may further be improved by more detailed investigations on the relationships between the lithographic process variables. The concept of photopatternable functional nanomaterials that are attainable via fluorous solution processing would be potentially useful in solution-processed organic electronic devices. In future research, the nanoparticles examined in this study should be mixed with larger BTO particles to prepare a bimodal distribution of ligand-stabilized particles. This approach is expected to enhance the packing density, as the smaller particles would fill the voids between the larger particles, yielding thin films with high dielectric constants.

## References

[1] Don Park Y., Lim J.A., Lee H.S., Cho K. (2007). Interface engineering in organic transistors. Mater. Today.

[2] Pei M., Ko J.S., Shin H., Cho M., Baek J., Kim G., Youk J.H., Yang H. (2018). Self-Assembly of Pentacene on Sub-nm Scale Surface Roughness-Controlled Gate Dielectrics. Macromol. Res..

[3] Li F.M., Nathan A., Wu Y.L., Ong B.S. (2007). Organic thin-film transistor integration using silicon nitride gate dielectric. Appl. Phys. Lett..

[4] Lee J., Kim J.H., Im S. (2003). Pentacene thin-film transistors with Al2O3+x gate dielectric films deposited on indium-tin-oxide glass. Appl. Phys. Lett..

[5] Halik M., Klauk H., Zschieschang U., Schmid G., Dehm C., Schütz M., Maisch S., Effenberger F., Brunnbauer M., Stellacci F. (2004). Low-voltage organic transistors with an amorphous molecular gate dielectric. Nature.

[6] Park Y.M., Desai A., Salleo A., Jimison L. (2013). Solution-Processable Zirconium Oxide Gate Dielectrics for Flexible Organic Field Effect Transistors Operated at Low Voltages. Chem. Mater..

[7] Sirringhaus H., Kawase T., Friend R.H., Shimoda T., Inbasekaran M., Wu W., Woo E.P. (2000). High-Resolution Inkjet Printing of All-Polymer Transistor Circuits. Science.

[8] Rogers J.A., Bao Z., Makhija A., Braun P. (1999). Printing Process Suitable for Reel-to-Reel Production of High-Performance Organic Transistors and Circuits. Adv. Mater..

[9] Yoon M.-H., Yan H., Facchetti A., Marks T.J. (2005). Low-Voltage Organic Field-Effect Transistors and Inverters Enabled by Ultrathin Cross-Linked Polymers as Gate Dielectrics. J. Am. Chem. Soc..

[10] Barber P., Balasubramanian S., Anguchamy Y., Gong S., Wibowo A., Gao H., Ploehn H.J., Zur Loye H.-C. (2009). Polymer Composite and Nanocomposite Dielectric Materials for Pulse Power Energy Storage. Materials.

[11] Su Y.R., Wang C.L., Xie W.G., Xie F.Y., Chen J., Zhao N., Xu J.B. (2011). Low-Voltage Organic Field-Effect Transistors (OFETs) with Solution-Processed Metal-Oxide as Gate Dielectric. ACS Appl. Mater. Interfaces.

[12] Balazs A.C., Emrick T., Russell T.P. (2006). Nanoparticle Polymer Composites: Where Two Small Worlds Meet. Science.

[13] Pujari S.P., Scheres L., Marcelis A.T.M., Zuilhof H. (2014). Covalent Surface Modification of Oxide Surfaces. Angew. Chem. Int. Edit..

[14] Kango S., Kalia S., Celli A., Njuguna J., Habibi Y., Kumar R. (2013). Surface modification of inorganic nanoparticles for development of organic-inorganic nanocomposites-A review. Prog. Polym. Sci..

[15] Gan Y., Cai Q.J., Li C.M., Yang H.B., Lu Z.S., Gong C., Chan-Park M.B. (2009). Solution-Prepared Hybrid-Nanoparticle Dielectrics for High-Performance Low-Voltage Organic Thin-Film Transistors. ACS Appl. Mater. Interfaces.

[16] Kim J., Lim S.H., Kim Y.S. (2010). Solution-Based TiO_2_−Polymer Composite Dielectric for Low Operating Voltage OTFTs. J. Am. Chem. Soc..

[17] Dang Z.-M., Wang H.-Y., Xu H.-P. (2006). Influence of silane coupling agent on morphology and dielectric property in BaTiO_3_/polyvinylidene fluoride composites. Appl. Phys. Lett..

[18] Bhattacharya S.K., Tummala R.R. (2000). Next generation integral passives: materials, processes, and integration of resistors and capacitors on PWB substrates. J. Mater. Sci. Mater. Electron..

[19] Zakhidov A.A., Lee J.-K., DeFranco J.A., Fong H.H., Taylor P.G., Chatzichristidi M., Ober C.K., Malliaras G.G. (2011). Orthogonal processing: A new strategy for organic electronics. Chem. Sci..

[20] Yang K., Huang X., Huang Y., Xie L., Jiang P. (2013). Fluoro-Polymer@BaTiO_3_ Hybrid Nanoparticles Prepared via RAFT Polymerization: Toward Ferroelectric Polymer Nanocomposites with High Dielectric Constant and Low Dielectric Loss for Energy Storage Application. Chem. Mater..

[21] Kim Y., Kim K.H., Lee A., Kim M.-S., Yoo B., Lee J.-K. (2017). Highly Fluorinated Polymer-Inorganic Nanoparticle Composites Processable with Fluorous Solvents. J. Nanosci. Nanotechnol..

[22] Cho S.-D., Lee J.-Y., Hyun J.-G., Paik K.-W. (2004). Study on epoxy/BaTiO_3_ composite embedded capacitor films (ECFs) for organic substrate applications. Mater. Sci. Eng. B.

[23] Uchino K., Sadanaga E., Hirose T. (1989). Dependence of the Crystal Structure on Particle Size in Barium Titanate. J. Am. Ceram. Soc..

[24] Waser R. (1997). Dielectric analysis of intergrated ceramic thin film capacitors. Integr. Ferroelectr..

[25] Arya P.R., Jha P., Subbanna G.N., Ganguli A.K. (2003). Polymeric citrate precursor route to the synthesis of nano-sized barium lead titanates. Mater. Res. Bull..

[26] Boulos M., Guillemet-Fritsch S., Mathieu F., Durand B., Lebey T., Bley V. (2005). Hydrothermal synthesis of nanosized BaTiO_3_ powders and dielectric properties of corresponding ceramics. Solid State Ion..

[27] Chen Z., Huang L., He J., Zhu Y., O’Brien S. (2011). New nonhydrolytic route to synthesize crystalline BaTiO_3_ nanocrystals with surface capping ligands. J. Mater. Res..

[28] Niederberger M., Garnweitner G. (2006). Organic reaction pathways in the nonaqueous synthesis of metal oxide nanoparticles. Chem. Eur. J..

[29] Zhang X., Chen H., Ma Y., Zhao C., Yang W. (2013). Preparation and dielectric properties of core–shell structural composites of poly(1H,1H,2H,2H-perfluorooctyl methacrylate)@BaTiO_3_ nanoparticles. Appl. Surf. Sci..

[30] Kim P., Jones S.C., Hotchkiss P.J., Haddock J.N., Kippelen B., Marder S.R., Perry J.W. (2007). Phosphonic Acid-Modified Barium Titanate Polymer Nanocomposites with High Permittivity and Dielectric Strength. Adv. Mater..

[31] Cai Q.J., Gan Y., Chan-Park M.B., Yang H.B., Lu Z.S., Li C.M., Guo J., Dong Z.L. (2009). Solution-Processable Barium Titanate and Strontium Titanate Nanoparticle Dielectrics for Low-Voltage Organic Thin-Film Transistors. Chem. Mater..

[32] De Roo J., Justo Y., De Keukeleere K., Van den Broeck F., Martins J.C., Van Driessche I., Hens Z. (2015). Carboxylic-Acid-Passivated Metal Oxide Nanocrystals: Ligand Exchange Characteristics of a New Binding Motif. Angew. Chem. Int. Ed..

[33] Kim H., Jo S.H., Jee J.-H., Han W., Kim Y., Park H.-H., Jin H.-J., Yoo B., Lee J.-K. (2015). Fluorous-inorganic hybrid dielectric materials for solution-processed electronic devices. New J. Chem..

[34] Studart A.R., Amstad E., Gauckler L.J. (2007). Colloidal Stabilization of Nanoparticles in Concentrated Suspensions. Langmuir.

[35] Mao Y., Felix N.M., Nguyen P.T., Ober C.K., Gleason K.K. (2006). Positive- and Negative-Tone CVD Polyacrylic Electron-Beam Resists Developable by Supercritical CO_2_. Chem. Vap. Depos..

[36] Zhu X., Wang J., Zhang Z., Zhu J., Zhou S., Liu Z., Ming N. (2008). Atomic-Scale Characterization of Barium Titanate Powders Formed by the Hydrothermal Process. J. Am. Ceram. Soc..

[37] Niederberger M., Garnweitner G., Pinna N., Antonietti M. (2004). Nonaqueous and Halide-Free Route to Crystalline BaTiO_3_, SrTiO_3_, and (Ba,Sr)TiO_3_ Nanoparticles via a Mechanism Involving C−C Bond Formation. J. Am. Chem. Soc..

[38] Takeuchi T., Tabuchi M., Ado K., Honjo K., Nakamura O., Kageyama H., Suyama Y., Ohtori N., Nagasawa M. (1997). Grain size dependence of dielectric properties of ultrafine BaTiO_3_ prepared by a sol-crystal method. J. Mater. Sci..

[39] Ashiri R. (2015). Obtaining a novel crystalline/amorphous core/shell structure in barium titanate nanocrystals by an innovative one-step approach. RSC Adv..

[40] Yin Y., Alivisatos A.P. (2005). Colloidal nanocrystal synthesis and the organic–inorganic interface. Nature.

[41] Takami S., Sato T., Mousavand T., Ohara S., Umetsu M., Adschiri T. (2007). Hydrothermal synthesis of surface-modified iron oxide nanoparticles. Mater. Lett..

[42] Ledwa K.A., Kępiński L. (2017). Dispersion of ceria nanoparticles on γ-alumina surface functionalized using long chain carboxylic acids. Appl. Surf. Sci..

[43] Kim Y., Lee S., Kim S. (2016). Preparation of Fluorous Solvent-Dispersed Fe_3_O_4_ Nanocrystals: Role of Oxygen in Ligand Exchange. Langmuir.

[44] Bain C.D., Troughton E.B., Tao Y.T., Evall J., Whitesides G.M., Nuzzo R.G. (1989). Formation of monolayer films by the spontaneous assembly of organic thiols from solution onto gold. J. Am. Chem. Soc..

[45] Tsutsumi K., Funaki Y., Hirokawa Y., Hashimoto T. (1999). Selective Incorporation of Palladium Nanoparticles into Microphase-Separated Domains of Poly(2-vinylpyridine)-block-polyisoprene. Langmuir.

[46] Devaraju N.G., Kim E.S., Lee B.I. (2005). The synthesis and dielectric study of BaTiO_3_/polyimide nanocomposite films. Microelectron. Eng..

[47] Arlt G., Hennings D., With G.d. (1985). Dielectric properties of fine-grained barium titanate ceramics. J. Appl. Phys..

[48] Horikawa T., Mikami N., Makita T., Tanimura J., Kataoka M., Sato K., Nunoshita M. (1993). Dielectric-Properties of (Ba, Sr)TiO_3_ Thin-Films Deposited by Rf-Sputtering. Jpn. J. Appl. Phys..

[49] Jayasundere N., Smith B.V. (1993). Dielectric-Constant for Binary Piezoelectric 0-3 Composites. J. Appl. Phys..

[50] Wada S., Yazawa A., Hoshina T., Kameshima Y., Kakemoto H., Tsurumi T., Kuroiwa Y. (2008). Preparation of barium titanate nanoparticle sphere arrays and their dielectric properties. IEEE Trans. Ultrason. Ferroelectr. Freq. Control..

[51] Todd M.G., Shi F.G. (2005). Complex permittivity of composite systems: a comprehensive interphase approach. IEEE Trans. Dielectr. Electr. Insul..

[52] Li J.Y., Zhang L., Ducharme S. (2007). Electric energy density of dielectric nanocomposites. Appl. Phys. Lett..

[53] Jung S.-H., Chuluunbandi K., Kim Y., Son J., Oh H.-T., Lee J.H., Lee J.-K., Beom-Hoan O. (2018). Investigation of degradation pathways of poly(semiperfluoroalkyl methacrylate) thin films induced by electron-beam irradiation. J. Polym. Sci. A Polym. Chem..

